# Evaluating the General Public's Knowledge of Malaria: A Nationally Representative Cross‐Sectional Study

**DOI:** 10.1002/mbo3.70151

**Published:** 2025-11-18

**Authors:** Husna Irfan Thalib, Sariya Khan, Mable Pereira, Faten Abouelmagd, Manal El Said

**Affiliations:** ^1^ General Medicine Practice Program Batterjee Medical College Jeddah Saudi Arabia; ^2^ School of Medicine Lincoln American University Georgetown Guyana; ^3^ Department of Medical Parasitology, Faculty of Medicine Sohag University Sohag Egypt; ^4^ Department of Microbiology, General Medicine Practice Program Batterjee Medical College Jeddah Saudi Arabia; ^5^ Department of Microbiology and Infection Prevention & Control Unit Theodor Bilharz Research Institute Giza Egypt

**Keywords:** education, knowledge, malaria, Tanzania, urbanization

## Abstract

Malaria is a life‐threatening disease caused by *Plasmodium*, transmitted through the bites of infected female *Anopheles* mosquitoes. Despite global efforts, malaria remains a major health burden in developing countries. In Tanzania, cultural beliefs and misconceptions often delay treatment, especially in rural areas. This article aims to evaluate the level of malaria knowledge among the Tanzanian population and identify demographic factors associated with disparities in awareness. This cross‐sectional study used secondary data from the 2021–2022 Tanzania Demographic and Health Survey, analyzing responses from 18,747 individuals aged 15 years and older. Data on malaria‐related knowledge, attitudes, and practices were collected through standardized questionnaires. Malaria knowledge varied significantly by age, gender, education, and location (*p* < 0.05). Awareness was highest among individuals aged 20–24 and lowest among those over 50. Urban residents had greater knowledge than their rural counterparts. Education was strongly linked to awareness, with those having secondary or higher education scoring better. Male‐headed households showed slightly higher knowledge levels. Media exposure and mobile phone ownership were also associated with increased malaria awareness. Bridging knowledge gaps through targeted education, digital tools, and improved rural health infrastructure is essential for effective malaria control in Tanzania.

## Graphical abstract

National survey of 18,747 Tanzanians reveals malaria knowledge disparities by age, education, gender, and residence. Urban, educated, and media‐exposed individuals showed higher awareness, while rural and older populations lagged. Targeted education, digital tools, and rural infrastructure improvements are vital to closing gaps and strengthening malaria prevention strategies.

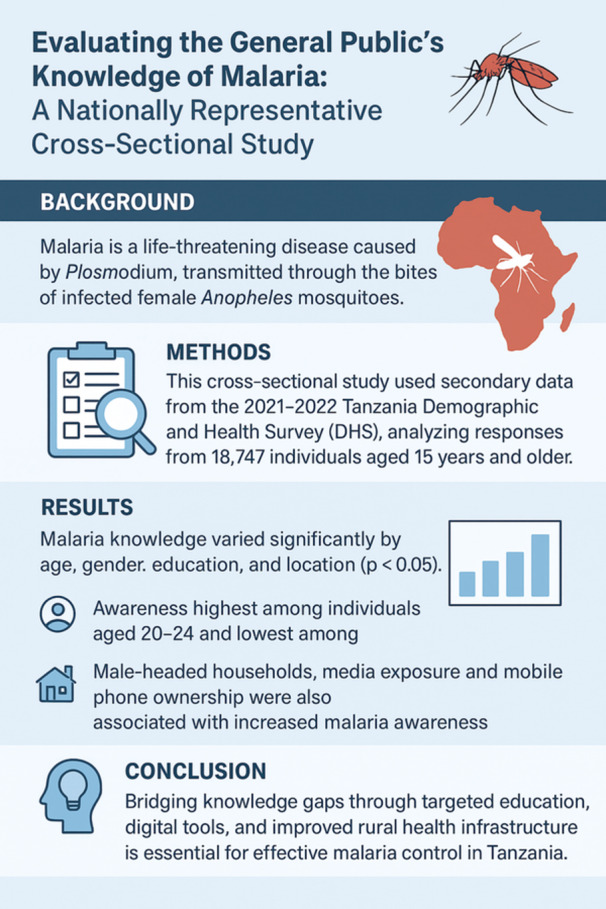

## 1 Introduction

Malaria is a life‐threatening disease caused by *Plasmodium* parasites, which are transmitted to humans through the bites of infected female *Anopheles* mosquitoes. Despite global efforts to eliminate the disease, malaria remains a critical public health issue, particularly in developing countries where it significantly contributes to morbidity and mortality (Zambare et al. 2019; Alonso and Tanner 2013). According to 2023 statistics by the World Health Organization (WHO), it was estimated that there were about 263 million cases of malaria worldwide (World Health Organization 2024). The burden of malaria is disproportionately higher in regions with limited healthcare resources, poor sanitation, and a lack of awareness about preventive measures. Socioeconomic and educational disparities also play a pivotal role in increasing vulnerability to malaria, as they hinder effective disease prevention and treatment. Many individuals in these regions lack the knowledge to recognize symptoms, understand modes of transmission, or adopt appropriate preventive measures, such as using insecticide‐treated nets (ITNs) and seeking timely medical intervention (Ricci 2012).

Malaria continues to pose a major public health challenge in both Tanzania and the broader sub‐Saharan African region. In Tanzania, over 95% of the population remains at risk of infection, and the disease is responsible for a disproportionate burden of morbidity and mortality, accounting for more than one‐third of deaths among children under five and up to one‐fifth among pregnant women (Mboera et al. 2007). Recent data from the Arusha Region alone indicated approximately 3.6 million malaria cases in 2023, underscoring the scale of transmission in localized high‐burden areas (Kołodziej et al. 2024). Across sub‐Saharan Africa, malaria is still deeply entrenched, with prevalence rates among children under five showing striking variability, ranging from as low as 0.7% in some regions to as high as 80.3% in others (Sarfo et al. 2023). The African region as a whole continues to carry the overwhelming global burden, accounting for 94% of malaria cases and 95% of malaria‐related deaths in 2023 (Bashir et al. 2025). These figures highlight both the persistent intensity of transmission and the urgent need for sustained and more effective control measures.

A growing body of evidence has demonstrated that insufficient public knowledge and persistent misconceptions about malaria remain critical barriers to effective control. In many rural communities, cultural beliefs and misinterpretations of symptoms delay care‐seeking and contribute to inappropriate treatment practices. Equally important, inadequate awareness of preventive strategies such as the necessity of sleeping under ITNs, the role of environmental management, and the value of prompt diagnostic testing has been consistently linked with higher disease prevalence and poorer outcomes (Wangdi et al. 2015; Ingabire et al. 2015). Such gaps in community understanding not only hinder timely treatment but also reduce adherence to control measures, thereby sustaining transmission cycles.

In this context, understanding the knowledge base of the general population is essential for guiding malaria control programs. By assessing malaria knowledge at a nationally representative level, it becomes possible to identify specific areas where misconceptions persist and to determine which demographic groups are most affected. This information is vital for tailoring health education initiatives, strengthening community engagement, and informing public health policies aimed at improving awareness and promoting preventive practices. Ultimately, bridging these knowledge gaps is crucial for enhancing the effectiveness of malaria control interventions and for accelerating progress toward reducing the overall burden of the disease.

The objective of this study is to evaluate the current level of knowledge about malaria among the general population of Tanzania and identify demographic and socioeconomic factors associated with knowledge disparities. This information is critical for strengthening public health initiatives aimed at reducing the malaria burden in endemic regions.

## 2 Materials and Methods

### 2.1 Study Design

This is a cross‐sectional study using secondary data from the Tanzania Demographic and Health Survey (DHS) conducted in 2021–2022. The DHS collects data on malaria‐related knowledge, attitudes, and practices as part of its national health assessment. Relevant variables for this study were selected from the survey data set for analysis.

### 2.2 Study Setting

Data were collected from rural and urban regions across the country during the 2021–2022 survey period.

### 2.3 Study Participants and Data Collection

The study includes a nationally representative sample of 18,747 individuals aged 15 years and older from diverse geographic and socioeconomic backgrounds. Data collection was conducted between 2021 and 2022 by trained survey teams using standardized questionnaires. Ethical approval for research was obtained through a proposal request to the DHS Program (https://dhsprogram.com/data/) (Ethical Data Code No. TZBR82FL).

### 2.4 Study Variables

The study focused on demographic variables, such as age, gender of household lead, residence type (urban/rural), level of education, and socioeconomic status, affecting access to mobile phones and the internet. Knowledge‐related variables included understanding of malaria prevention methods and treatment‐seeking behavior.

### 2.5 Statistical Analysis

Data were organized using Microsoft Excel 2020 and exported for analysis using IBM SPSS Statistics version 25. Descriptive statistics were used to summarize demographic characteristics and knowledge levels. Chi‐square tests were performed to assess associations between demographic variables and malaria knowledge. A *p* value of < 0.05 was considered statistically significant.

## 3 Results

The study included 18,747 participants with a majority from rural areas (71.5%, *n* = 13,400) and a smaller proportion from urban regions (28.5%, *n* = 5347) as demonstrated in Table 1. Most participants were aged 35–39 years (21.5%, *n* = 4033), with the least representation in the 15–19 age group (1.3%, *n* = 236). Regarding education, a significant number had only primary education (57.7%, *n* = 10,817), while only 0.6% (*n* = 106) had higher education. Ownership of mobile phones was limited to 35.5% (*n* = 6652), and 90% of participants reported never using the internet.

**Table 1:** Demographic characteristics of study participants.

Characteristic	No. of participants (*N*)	Percentage (%)
*Age groups*		
15–19	236	1.3
20–24	1412	7.53
25–29	2626	14
30–34	3166	16.88
35–39	4033	21.51
40–44	3507	18.7
45–49	3767	20.09
*Gender of household lead*		
Male	13,698	73.06
Female	5049	26.93
*Type of place of residence*		
Urban	5347	28.52
Rural	13,400	71.47
*Education level*		
No education	4479	23.89
Primary	10,817	57.69
Secondary	3345	17.84
Higher	106	0.56
*Owns a mobile phone*		
Yes	6652	35.4
No	12,095	64.51
*Use of internet*		
Never	16,891	90.09
Yes, last 12 months	1656	8.83
Yes, before last 12 months	200	1.06
*Visited by a healthcare worker*		
Yes	16,853	89.89
No	1894	10.10
Total	18,747	100

Knowledge about malaria varied significantly across age groups (*p* < 0.05), as shown in Table 2. Younger participants aged 20–24 years showed the highest agreement with statements like the importance of sleeping under a net every night (96.8%) and ensuring malaria testing (90.9%). In contrast, older groups, such as those aged 45–49 years, demonstrated relatively lower levels of agreement, highlighting potential gaps in knowledge with increasing age.

**Table 2:** Correlation of malaria knowledge statements with age groups.

	Response	Age groups								*p* value
Statement		15–19 (*N* = 236)	20–24 (*N* = 1412)	25–29 (*N* = 2626)	30–34 (*N* = 3166)	35–39 (*N* = 4033)	40–44 (*N* = 3507)	45–49 (*N* = 3767)	Total (*N* = 18,747)	
Can easily protect self and children from malaria	Agree	213 (90.3%)	1320 (93.5%)	2438 (92.8%)	2935 (92.7%)	3772 (93.5%)	3257 (92.9%)	3482 (92.4%)	17,417 (92.9%)	0.001*
	Disagree	19 (8.1%)	78 (5.5%)	187 (7.1%)	226 (7.1%)	243 (6.0%)	236 (6.7%)	242 (6.4%)	1231 (6.6%)	
	Uncertain	4 (1.7%)	14 (1.0%)	1 (0.0%)	5 (0.2%)	18 (0.4%)	14 (0.4%)	43 (1.1%)	99 (0.5%)	
Important to sleep under a net every night	Agree	222 (94.1%)	1367 (96.8%)	2516 (95.8%)	3032 (95.8%)	3857 (95.6%)	3357 (95.7%)	3617 (96.0%)	17,966 (95.8%)	0.001*
	Disagree	12 (5.1%)	35 (2.5%)	107 (4.1%)	134 (4.2%)	176 (4.4%)	147 (4.2%)	124 (3.3%)	735 (3.9%)	
	Uncertain	2 (0.8%)	10 (0.7%)	3 (0.1%)	0 (0.0%)	0 (0.0%)	3 (0.1%)	28 (0.7%)	46 (0.2%)	
Pregnant women are at risk to malaria	Agree	197 (83.5%)	1205 (85.3%)	2331 (88.8%)	2849 (90.0%)	3610 (89.5%)	3127 (89.2%)	3372 (89.5%)	16,691 (89.0%)	0.001*
	Disagree	26 (11.0%)	115 (8.1%)	195 (7.4%)	228 (7.2%)	296 (7.3%)	219 (6.2%)	210 (5.6%)	1289 (6.9%)	
	Uncertain	13 (5.5%)	92 (6.5%)	100 (3.8%)	89 (2.8%)	127 (3.1%)	161 (4.6%)	185 (4.9%)	767 (4.1%)	
Can easily get treatment for children for malaria	Agree	223 (94.5%)	1264 (89.5%)	2430 (92.5%)	2938 (92.8%)	3687 (91.4%)	3242 (92.4%)	3522 (93.5%)	17,306 (92.3%)	0.001*
	Disagree	11 (4.7%)	116 (8.2%)	172 (6.5%)	213 (6.7%)	303 (7.5%)	242 (6.9%)	185 (4.9%)	1242 (6.6%)	
	Uncertain	2 (0.8%)	32 (2.3%)	24 (0.9%)	15 (0.5%)	43 (1.1%)	23 (0.7%)	60 (1.6%)	199 (1.1%)	
To ensure malaria, need to test	Agree	217 (91.9%)	1284 (90.9%)	2463 (93.8%)	2985 (94.3%)	3823 (94.8%)	3263 (93.0%)	3567 (94.7%)	17,602 (93.9%)	0.001*
	Disagree	15 (6.4%)	104 (7.4%)	117 (4.5%)	144 (4.5%)	159 (3.9%)	207 (5.9%)	161 (4.3%)	907 (4.8%)	
	Uncertain	4 (1.7%)	24 (1.7%)	46 (1.8%)	37 (1.2%)	51 (1.3%)	37 (1.1%)	39 (1.0%)	238 (1.3%)	
Important to take entire course of treatment	Agree	230 (97.5%)	1366 (96.7%)	2518 (95.9%)	3067 (96.9%)	3902 (96.8%)	3394 (96.8%)	3692 (98.0%)	18,169 (96.9%)	0.001*
	Disagree	5 (2.1%)	35 (2.5%)	98 (3.7%)	81 (2.6%)	111 (2.8%)	112 (3.2%)	68 (1.8%)	510 (2.7%)	
	Uncertain	1 (0.4%)	11 (0.8%)	10 (0.4%)	18 (0.6%)	20 (0.5%)	1 (0.0%)	7 (0.2%)	68 (0.4%)	

* Denotes significant *p* < 0.05.

Male‐headed households demonstrated slightly higher malaria knowledge than female‐headed households. For example, 95.6% of males agreed on the importance of sleeping under a net compared with 96.4% of females (*p* = 0.011), as indicated in Table 3. The differences in other statements, such as recognizing malaria risks for pregnant women, were not statistically significant (*p* > 0.05).

**Table 3:** Correlation of malaria knowledge statements with gender of household lead.

	Response	Gender of household lead		Total (*N* = 18,747)	*p* value
Statement		Male (*N* = 13,698)	Female (*N* = 5049)		
Can easily protect self and children from malaria	Agree	12,750 (93.1%)	4667 (92.4%)	17,417 (92.9%)	0.309
	Disagree	878 (6.4%)	353 (7.0%)	1231 (6.6%)	
	Uncertain	70 (0.5%)	29 (0.6%)	99 (0.5%)	
Important to sleep under a net every night	Agree	13,096 (95.6%)	4870 (96.5%)	17,966 (95.8%)	0.011*
	Disagree	562 (4.1%)	173 (3.4%)	735 (3.9%)	
	Uncertain	40 (0.3%)	6 (0.1%)	46 (0.2%)	
Pregnant women are at risk to malaria	Agree	12,216 (89.2%)	4475 (88.6%)	16,691 (89.0%)	0.552
	Disagree	927 (6.8%)	362 (7.2%)	1289 (6.9%)	
	Uncertain	555 (4.1%)	212 (4.2%)	767 (4.1%)	
Can easily get treatment for children for malaria	Agree	12,642 (92.3%)	4664 (92.4%)	17,306 (92.3%)	0.001*
	Disagree	889 (6.5%)	353 (7.0%)	1242 (6.6%)	
	Uncertain	167 (1.2%)	32 (0.6%)	199 (1.1%)	
To ensure malaria, need to test	Agree	12,882 (94.0%)	4720 (93.5%)	17,602 (93.9%)	0.033*
	Disagree	633 (4.6%)	274 (5.4%)	907 (4.8%)	
	Uncertain	183 (1.3%)	55 (1.1%)	238 (1.3%)	
Important to take entire course of treatment	Agree	13,233 (96.6%)	4936 (97.8%)	18,169 (96.9%)	0.001*
	Disagree	416 (3.0%)	94 (1.9%)	510 (2.7%)	
	Uncertain	49 (0.4%)	19 (0.4%)	68 (0.4%)	

* Denotes significant *p* < 0.05.

Participants from rural areas were slightly less knowledgeable compared with their urban counterparts across most statements. For instance, 94.3% of urban residents agreed on the importance of using mosquito nets, compared with 95.4% of rural residents (*p* < 0.05), as shown in Table 4. However, rural participants were slightly more likely to express uncertainty about malaria prevention methods.

**Table 4:** Correlation of malaria knowledge statements with the type of place of residence.

	Response	Type of place of residence		Total (*N* = 18,747)	*p* value
Statement		Urban (*N* = 5347)	Rural (*N* = 13,400)		
Can easily protect self and children from malaria	Agree	5028 (94.0%)	12,389 (92.4%)	17,417 (92.9%)	0.001*
	Disagree	301 (5.6%)	930 (6.9%)	1231 (6.6%)	
	Uncertain	18 (0.3%)	81 (0.6%)	99 (0.5%)	
Important to sleep under a net every night	Agree	5181 (96.9%)	12,785 (95.4%)	17,966 (95.8%)	0.001*
	Disagree	161 (3.0%)	574 (4.3%)	735 (3.9%)	
	Uncertain	5 (0.1%)	41 (0.3%)	46 (0.2%)	
Pregnant women are at risk to malaria	Agree	4801 (89.8%)	11,890 (88.7%)	16,691 (89.0%)	0.058
	Disagree	331 (6.2%)	958 (7.2%)	1289 (6.9%)	
	Uncertain	215 (4.0%)	552 (4.1%)	767 (4.1%)	
Can easily get treatment for children for malaria	Agree	5080 (95.0%)	12,226 (91.2%)	17,306 (92.3%)	0.001*
	Disagree	220 (4.1%)	1022 (7.6%)	1242 (6.6%)	
	Uncertain	47 (0.9%)	152 (1.1%)	199 (1.1%)	
To ensure malaria, need to test	Agree	5171 (96.7%)	12,431 (92.7%)	17,602 (93.9%)	0.001*
	Disagree	123 (2.3%)	784 (5.8%)	907 (4.8%)	
	Uncertain	53 (1.0%)	185 (1.4%)	238 (1.3%)	
Important to take entire course of treatment	Agree	5249 (98.2%)	12,920 (96.4%)	18,169 (96.9%)	0.001*
	Disagree	92 (1.7%)	418 (3.1%)	510 (2.7%)	
	Uncertain	6 (0.1%)	62 (0.5%)	68 (0.4%)	

* Denotes significant *p* < 0.05.

**Figure 1:**
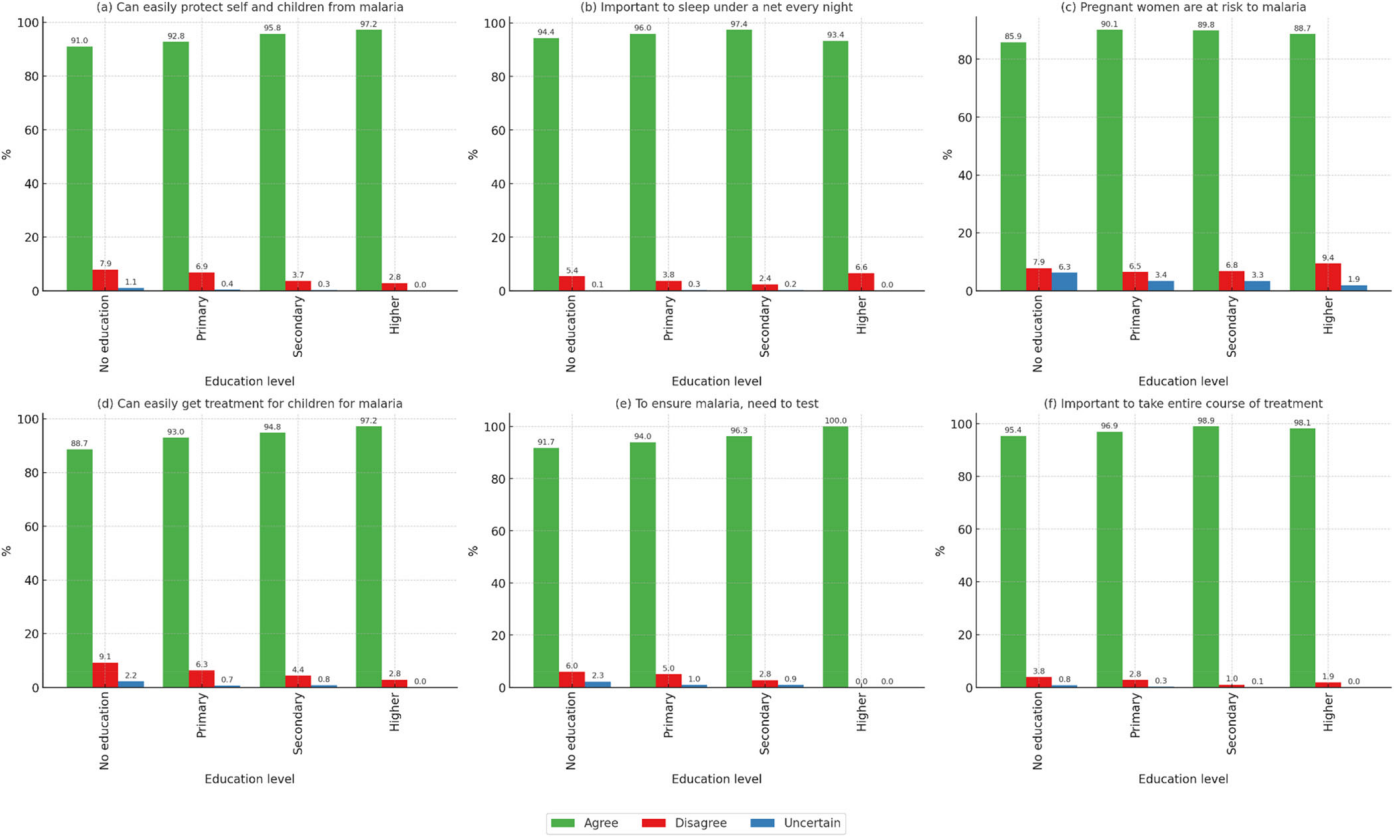
Correlation of malaria knowledge statements with education level. *All statements denote significant *p* < 0.05. (a) Can easily protect self and children from malaria, (b) important to sleep under a net every night, (c) pregnant women are at risk to malaria, (d) can easily get treatment for children for malaria, (e) to ensure malaria, need to test, and (f) important to take entire course of treatment.

Education level significantly impacted malaria knowledge (*p* < 0.05). Participants with secondary or higher education showed near‐universal agreement on preventive measures like using mosquito nets (98.2%) and completing malaria treatment courses (97.9%), as shown in Figure 1. In contrast, those with no education exhibited lower agreement and higher levels of uncertainty.

**Figure 2:**
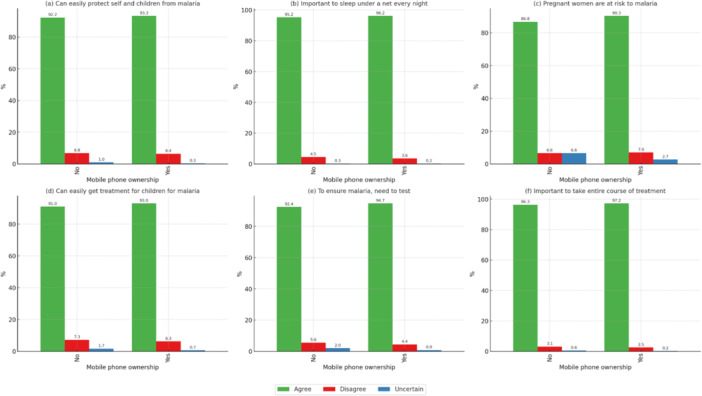
Correlation of malaria knowledge statements with “owns a mobile.” *All statements denote significant *p* < 0.05. (a) Can easily protect self and children from malaria, (b) important to sleep under a net every night, (c) pregnant women are at risk to malaria, (d) can easily get treatment for children for malaria, (e) to ensure malaria, need to test, and (f) important to take entire course of treatment.

Mobile phone ownership correlated with improved malaria knowledge (*p* < 0.05). For instance, 93.2% of mobile phone owners agreed on the need to ensure malaria testing, compared with 91.3% of nonowners. Access to phones likely facilitated greater exposure to health information and malaria prevention methods, as shown in Figure 2.

**Figure 3:**
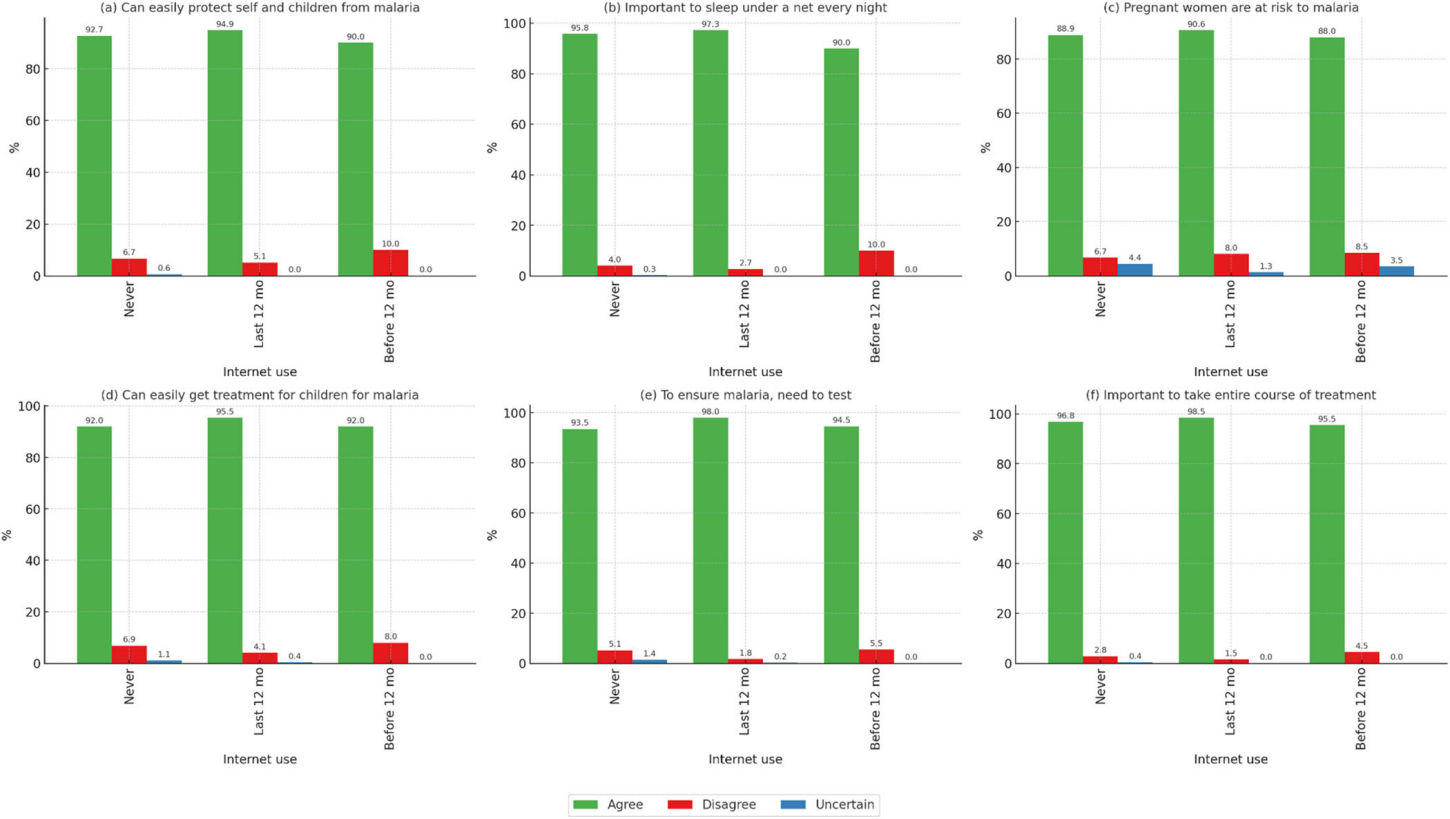
Correlation of malaria knowledge statements with use of internet. *All statements denote significant *p* < 0.05. (a) Can easily protect self and children from malaria, (b) important to sleep under a net every night, (c) pregnant women are at risk to malaria, (d) can easily get treatment for children for malaria, (e) to ensure malaria, need to test, and (f) important to take entire course of treatment.

Internet users demonstrated significantly higher knowledge about malaria (*p* < 0.05). Participants who had accessed the internet in the past 12 months showed 97.3% agreement on using mosquito nets and completing malaria treatment, compared with 96.6% among nonusers, as demonstrated in Figure 3.

**Figure 4:**
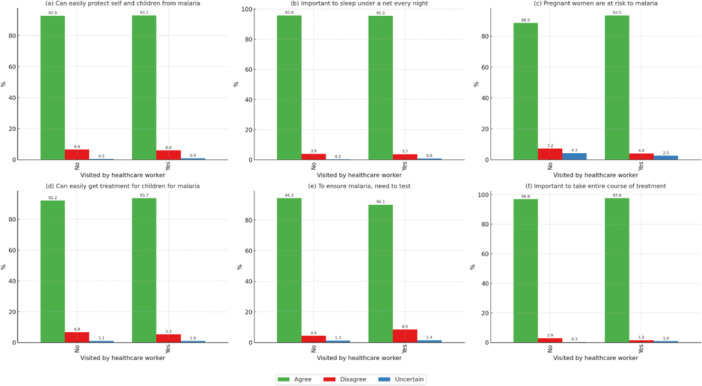
Correlation of malaria knowledge statements with visited by healthcare worker who talked about malaria. *All statements denote significant *p* < 0.05. (a) Can easily protect self and children from malaria, (b) important to sleep under a net every night, (c) pregnant women are at risk to malaria, (d) can easily get treatment for children for malaria, (e) to ensure malaria, need to test, and (f) important to take entire course of treatment.

Participants visited by healthcare workers showed significantly better knowledge (*p* < 0.05). Those visited (97.6%) agreed on the importance of completing malaria treatment courses, compared with 96.9% of those not visited. This emphasizes the role of direct community health engagement in improving malaria awareness, as shown in Figure 4.

## 4 Discussion

The study identifies notable gaps in malaria knowledge across demographic and socioeconomic variables of age, education, gender, geographical location, and access to technology (Table 1). The results call for focused public health campaigns to eliminate knowledge gaps among older people, those with less education, and rural dwellers. Among the key findings is the inverse relation between age and malaria awareness, where the lowest age group of respondents (20–24 years) had the greatest awareness about prevention, while older respondents (45–49 years) expressed lower levels of consensus (Table 2). This may suggest that younger generations are likely to receive more exposure to public health communication, school health education, and internet resources for improving malaria awareness. On the other hand, older groups would rely on classic information or outdated knowledge that could delay the adoption of effective preventive measures. These findings are different from what was observed in a study conducted in India in 2007, where it was found that age was not a significant predictor for knowledge of malaria prevention techniques (Sharma et al. 2007). However, our findings are compliant with another study conducted in Tanzania in 2014, where it was found that those in the age group 30–49 years exhibited higher levels of knowledge as compared with those 50 and above (Spjeldnæs et al. 2014).

Educational level was also among the strongest predictors of malaria knowledge, with secondary and tertiary levels having near‐universal concordance for preventive measures and persons without education being less concordant and uncertain (Figure 1). This conforms to literature, which indicates that higher education contributes to better health literacy, through which people gain more knowledge concerning disease prevention and cure (Dike et al. 2006; Kouamé et al. 2022). Public health initiatives should thus accord high priority to expanding malaria education among those with fewer years of formal education, perhaps through sensitization campaigns at the community level or pictorial media that deconstruct complicated messages. Gender differences were also evident, with male‐headed households recording a marginally higher level of knowledge of malaria than female‐headed households. This might be attributed to variations in educational attainment, decision‐making status, or access to health information among men and women. It was observed that our findings were consistent with a study conducted in Bangladesh in 2009, where it was concluded that the knowledge regarding prevention techniques was lower among women (Ahmed et al. 2009). Another study in Burkin Faso in 2017 also found similar results (Yaya et al. 2017). Because women are usually the primary caregivers, educating them about malaria is essential to successful prevention and treatment compliance within the home. Interventions that are culturally relevant must be crafted in an effort to break through this imbalance by ensuring equal access to health education by both males and females (Table 3).

Geographic variation highlights important challenges in malaria education, particularly for rural communities, whose knowledge levels were slightly lower than those of urban respondents (Table 4). Rural populations often face barriers, such as limited access to healthcare facilities, reduced availability of educational materials, and fewer opportunities to engage with technology. These constraints likely contribute to the observed gaps in awareness. However, this finding does not appear to be universal. A study from Enugu, Nigeria, reported that rural and urban communities had almost the same level of awareness (Oguonu et al. 2005), suggesting that local context and the strength of health systems strongly influence whether such disparities are observed.

Addressing these gaps requires a combination of strategies. Community health outreach activities need to be expanded and better coordinated, especially in hard‐to‐reach areas. Scaling up mobile health (mHealth) interventions is another promising approach, as these have been shown to deliver health information effectively at low cost. Involving local healthcare workers in health promotion efforts also ensures that accurate information reaches households through trusted channels.

The role of technology was particularly evident in this study. Both mobile phone ownership and recent internet use were positively associated with greater malaria knowledge (Figures 2 and 3). Individuals who owned mobile phones or had accessed the internet in the past 12 months were more likely to be aware of preventive strategies, such as consistent use of ITNs and the need to complete treatment courses. This pattern aligns with the findings of a systematic review conducted in sub‐Saharan Africa in 2014, which concluded that mobile health interventions improved awareness and supported preventive behaviors (Brinkel et al. 2014). These observations indicate that technology provides a valuable opportunity for public health campaigns, especially in contexts where traditional health infrastructure is limited.

The policy relevance of these findings is clear. Malaria education should be prioritized among groups at higher risk of being left behind, including rural populations and individuals with lower literacy levels. Technology‐based campaigns that use simple, accessible messaging can help overcome barriers to communication, while investments in rural health infrastructure can provide the necessary support for these efforts. These results are consistent with the findings of a meta‐analysis published in 2023, which highlighted that community‐based malaria prevention programs are most effective when interventions are directed at specific demographic groups (Onyinyechi et al. 2023).

Our study has limitations that must be acknowledged. The cross‐sectional design limits the ability to draw conclusions about cause‐and‐effect relationships between demographic characteristics and malaria knowledge (Lee et al. 2022). For instance, while internet use was associated with higher knowledge, it cannot be determined whether internet access led to improved knowledge or whether more knowledgeable individuals were more likely to seek information online. In addition, reliance on self‐reported responses introduces the possibility of bias, as participants may have overstated their knowledge to provide socially desirable answers. Finally, the study did not account for prior episodes of malaria, either personal or within the household, which may strongly influence knowledge levels. Future studies should adopt longitudinal designs to track changes in malaria knowledge over time and to assess the long‐term impact of targeted interventions. Including prior malaria experience as an explanatory factor would also provide a more complete understanding of how awareness develops and changes in different communities.

## 5 Conclusion

This study identifies the effect of age, education, gender, geographic location, and access to technology on malaria awareness. Closing awareness gaps by focused education, e‐interventions, and improved rural health facilities is crucial to enhancing malaria prevention and control activities. Future interventions must focus on the utilization of mobile technology and community‐based interventions to close awareness gaps and enhance equitable access to malaria information.

## Author Contributions

**Husna Irfan Thalib:** conceptualization, study design, questionnaire development, data collection, formal analysis, writing – original draft. **Sariya Khan:** data curation, literature review, writing – review and editing. **Faten Abouelmagd:** supervision, conceptualization, methodology, investigation, data interpretation, writing – review and editing. **Manal El Said:** methodology, supervision, formal analysis, writing – review and editing. All authors have read and approved the final manuscript and agree to be accountable for all aspects of the work.

## Ethics Statement

The authors have nothing to report.

## Conflicts of Interest

The authors declare no conflicts of interest.

## Data Availability

The data that support the findings of this study are available on request from the corresponding author. The data are not publicly available due to privacy or ethical restrictions.
